# *In Vitro* Anti-HMPV Activity of Meroditerpenoids from Marine Alga *Stypopodium zonale* (Dictyotales)

**DOI:** 10.3390/molecules16108437

**Published:** 2011-10-10

**Authors:** Gabriella Mendes, Angélica Ribeiro Soares, Lorena Sigiliano, Fernanda Machado, Carlos Kaiser, Nelilma Romeiro, Lísia Gestinari, Norma Santos, Maria Teresa Villela Romanos

**Affiliations:** 1 Laboratório Experimental de Drogas Antivirais e Citotóxicas (LEDAC), Departamento de Virologia do, Instituto de Microbiologia Paulo de Góes, Universidade Federal do Rio de Janeiro (UFRJ), CCS, Bloco I, Caixa Postal 68040, 21941-590 Rio de Janeiro, RJ, Brasil; 2 Laboratório de Viroses Respiratórias, Entéricas e Oculares, Departamento de Virologia do Instituto de Microbiologia Paulo de Góes, Universidade Federal do Rio de Janeiro (UFRJ), CCS, Bloco I, Caixa Postal 68040, 21941-590 Rio de Janeiro, RJ, Brasil; 3 Grupo de Produtos Naturais de Organismos Aquáticos (GPNOA), Núcleo em Ecologia e Desenvolvimento Socioambiental de Macaé (NUPEM), Universidade Federal do Rio de Janeiro (UFRJ), CCS, Bloco I, Caixa Postal 68040, 21941-590 Rio de Janeiro, RJ, Brasil; 4 Instituto de Química, Universidade Federal do Rio de Janeiro (UFRJ), CCS, Bloco I, Caixa Postal 68040, 21941-590 Rio de Janeiro, RJ, Brasil

**Keywords:** HMPV, alga, antiviral, meroditerpenoids, Dictyotaceae

## Abstract

In this paper, we evaluated the antiviral activity against HMPV replication of crude extract of the marine algae *Stypopodium zonale* and of two meroditerpenoids obtained from it, atomaric acid and epitaondiol, and a methyl ester derivative of atomaric acid. Their selectivity indexes were 20.78, >56.81, 49.26 and 12.82, respectively. Compared to ribavirin, the substances showed a relatively low cytotoxicity on LLC-MK2 cells, with a significant antiviral activity, inhibiting at least 90% of viral replication *in vitro*, which demonstrates the potential of these marine natural products to combat infections caused by HMPV *in vitro*.

## 1. Introduction

Human metapneumovirus (HMPV) is a member of the *Paramyxovidae* family, along with the related respiratory syncytial virus (RSV) and parainfluenza virus (PIV) [1]. HMPV is an important worldwide agent of severe respiratory disease leading to significant morbidity in infants and other special populations, including immunocompromised and elderly patients [2,3,4,5,6,7,8,9,10]. Serological studies have revealed that HMPV seropositivity is almost universal by the age of 5 [1,11]. In Brazil, the burden of HMPV infections has been demonstrated by several scientists across the country [12,13,14,15,16,17].

Other than for influenza virus, antiviral therapy for respiratory viruses has not shown much potential. Several agents have been evaluated for their effect on HMPV replication *in vitro* or in animal models [5]. Ribavirin and pooled human immunoglobulin inhibit HMPV and RSV replication equally in cell culture; however the effectiveness of ribavirin therapy for RSV is limited, at best, and remains a controversial issue [18]. Palivizumab and other chemotherapeutics directed at the F protein of RSV are not active against HMPV [18]. NMSO_3_, a sulfated sialyl lipid known to inhibit RSV replication in cell culture and in the cotton rat model, has also been shown to inhibit HMPV replication, syncytia formation, and cell-to-cell virus spread in culture [19]. None of these compounds have been systematically tested in humans for the treatment of HMPV infection, although a case report describes an apparently successful treatment with ribavirin of a patient who had undergone a lung transplant and had a severe HMPV infection [20].

The brown seaweed *Stypopodium zonale* (Lamouroux) Papenfuss is found abundantly along the Brazilian coast and is known to produce meroditerpenes, which are compounds of mixed biosynthesis [21,22]. Several of them display diverse and useful biological properties, and some have had their pharmacological activity, including antiviral activity, described in the literature [22,23,24].

Due to its high sensitivity, that increases the quality of the assays, the real time PCR methodology has recently been applied to evaluate antiviral drugs, based on the quantitative detection of DNA in infected cells [25,26,27,28,29,30].

Thus, the main purpose of this study was to evaluate the antiviral activity in the HMPV replication assay by real time PCR of: (i) the crude extract of the alga *S. zonale*; (ii) two meroditerpenoids previously isolated from *S. zonale*, atomaric acid and epitaondiol; and (iii) a methyl ester derivative of atomaric acid.

## 2. Results and Discussion

**Figure 1:**
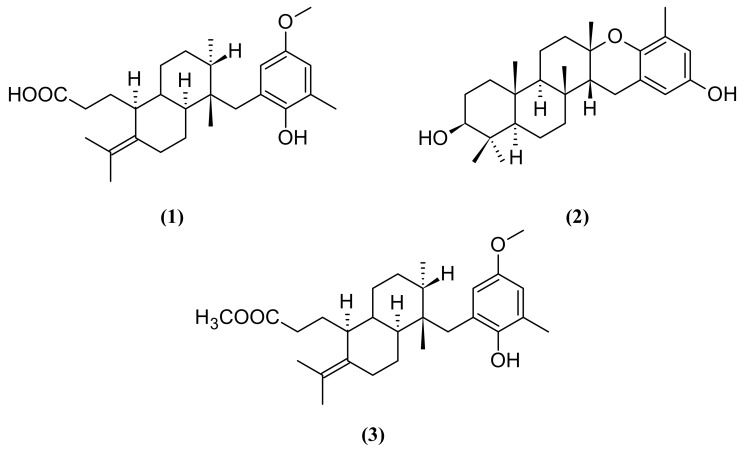
Structures of atomaric acid (**1**), epitaondiol (**2**) and the methyl ester of atomaric acid **3**.

In the present study, we investigated the anti-HMPV activity of the crude extract and some secondary metabolites from *S. zonale*. The ^1^H-NMR spectrum of the crude extract revealed the presence of atomaric acid (**1**) as the principal component. The crude extract and the methylated crude extract were individually chromatographed on silica gel to afford the known meroditerpenes atomaric acid (**1**), epitaondiol (**2**) and the methyl ester derivative of the atomaric acid (**3**) (Figure 1) which has been obtained using a semi-synthetic derivatization approach in order to evaluate the effect of increase in the non-polar character of the compounds, and represents a usual chemical modification strategy.

Cytotoxicity assays were performed before the antiviral tests, in order to avoid false results. After establishing the cytotoxicity of each substance we have chosen to use the concentration in which 100% of the cells were viable. For the antiviral experiments we used concentrations of 12.5 µg/mL for the crude extract of *S. zonale*, epitaondiol and the methyl ester of atomaric and 25 µg/mL for atomaric acid.

The crude extract and epitaondiol (**2**) displayed significant toxicity for LLC-MK2 cells, with CC_50_ values of 31 µg/mL and 50 µg/mL, respectively. Such toxicity is not unusual for compounds extracted from the genus *Stypopodium*. For instance, meroditerpenoids isolated by Sabry *et al.* [31] from this alga displayed strong neurotoxic activity towards the murine Neuro-2a neuroblastoma cell line. Moreover, *in vitro* screening of the crude dichloromethane-chloroform extract of *S. zonale* showed its cytotoxicity against human C32 cells (human amelanotic melanoma cell line) using the sulphorhodamine B assay [32]. In our study, atomaric acid showed a CC_50_ value higher than 200 µg/mL but, on the other hand, its methyl ester showed significant toxicity for the cell culture, with a CC_50_ value of 34 µg/mL.

**Figure 2:**
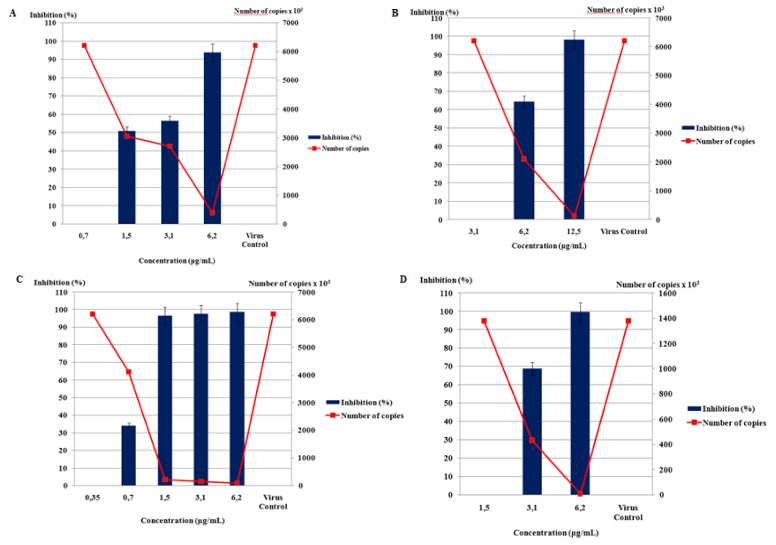
Kinetics of inhibition of the four compounds tested against HMPV. (**A**) *S. zonale* crude extract; (**B**) atomaric acid; (**C**) epitaondiol; (**D**) methyl ester of atomaric acid.

The evaluation of the antiviral activity, which involved the comparison of the number of genomic RNA copies obtained in the presence of the three compounds and the one obtained from the viral control, resulted in inhibition rates of 97.7% for the *S. zonale* crude extract, 99.4% for atomaric acid (**1**), and >99.99% for epitaondiol (**2**) and for the methyl ester derivative **3** obtained by derivatization of atomaric acid. Figure 2 shows diagrams correlating percentages of inhibition and the genome equivalents measured by real time RT-PCR of the virus with/without treatment (virus control). The results show that the four compounds displayed a dose-dependent activity, as the viral inhibition increases together with the concentration. Table 1 shows the correlation between cytotoxicity and antiviral activity. The ED_50_ values were obtained by correlating the concentration with the percentage of inhibition. The selectivity indexes were obtained by the correlation between CC_50_ and ED_50_. A previous study has demonstrated the antiviral activity of the meroditerpenes atomaric acid and epitaondiol against *herpes simplex virus* type 1 (HSV-1), yet in the same study this secondary metabolite showed no activity against human immunodeficiency virus (HIV) [22].

**Figure 3:**
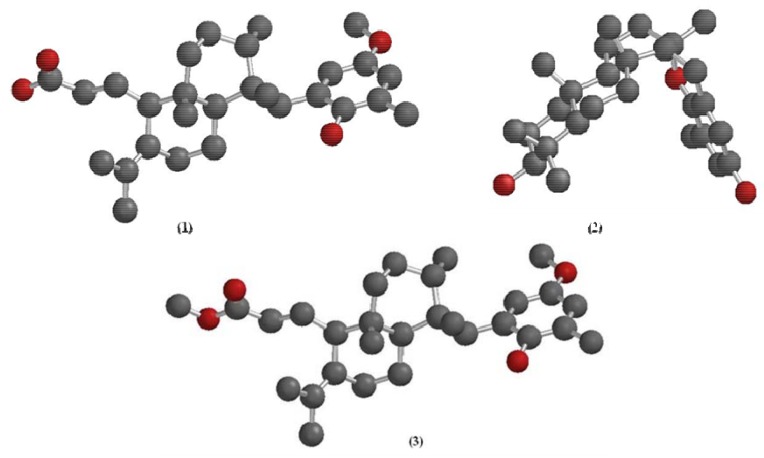
Energy-minimized 3D structures of atomaric acid (**1**), epitaondiol (**2**) and atomaric acid methyl ester (**3**).

In order to evaluate the anti-HMPV activity of the substances, LLC-MK2 cell monolayers were treated with different concentrations of the crude extract and the three substances, separately. A dose-dependent response curve was observed for all the substances, meaning that the lower the concentration, the lower the activity. We could also calculate the ED_50_ of the *S. zonale* crude extract (1.48 µg/mL) and substances **1**-**3** (Figure 1). Epitaondiol (**2**) displayed better anti-HMPV activity (1.01 µg/mL) followed by the methyl ester derivative **3** (2.66 µg/mL) and its precursor, atomaric acid (**1**) (3.52 µg/mL) (Table 1). Different structural characteristics were observed between the compounds. The increased lipophilicity caused by the hydroxyl substitution for the methoxyl group [logP values of 7.04 were calculated for the methyl ester **3** and 3.37 for atomaric acid (**1**), in its ionized form] may furnish some explanation for the difference on the activity shown by the methyl ester in comparison to atomaric acid, probably due to enhanced cell permeability. However, this substitution also enhanced the toxicity of the compound and further hydrolysis of the ester derivative cannot be discarded. Moreover, the meroditerpenoid epitaondiol, a side-chain cyclized tocopherol-like derivative, has molecular features in its skeleton that differ significantly from the atomaric acid-related compounds that may be responsible for the biological activity presented herein. Epitoandiol (**2**), for instance, has a more conformationally restricted structure than the other compounds due the presence of the two additional fused rings. Also, although it has a similar calculated logP compared to the methyl ester of atomaric acid (6.02 *vs.* 7.04), it has a smaller solvent-accessible surface area (493.7 *vs.* 663.4 and 714.2 for atomaric acid and the methyl ester, respectively). This is due to a more “closed” equilibrium conformation shown by this compound (Figure 3) in comparison to extended and more solvent-accessible conformations of atomaric acid and its ester derivative, which may also be responsible for a differential molecular recognition of these marine natural products by protein targets.

## 3. Mechanisms of Action

**Figure 4:**
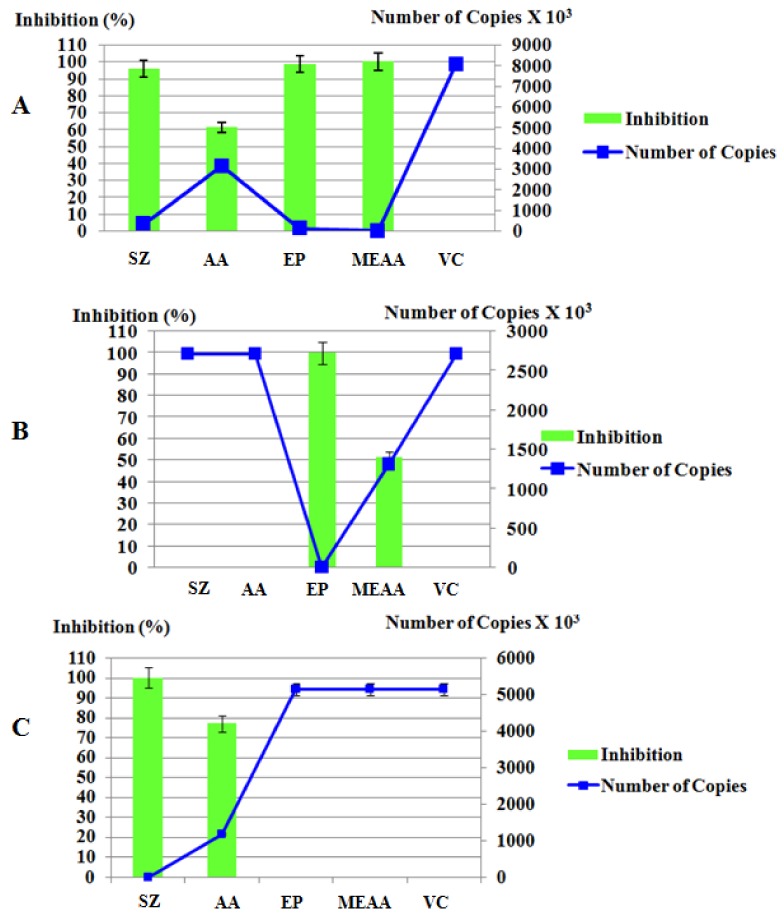
Correlation between viral genome equivalents and percentage of inhibition during different stages of viral replication. (**A**) Virucidal activity; (**B**) Penetration activity; (C) Post-penetration.

The mechanism of action differed according to the substance analyzed. The *S. zonale* crude extract and atomaric acid were capable of interacting with the viral particles outside the cells and preventing the infection of the cell cultures. Both had no activity on the cellular receptors or during the penetration of the viral particles, but were capable of inhibiting the post-penetration stage. Nevertheless, atomaric acid was less active than the crude extract of *S. zonale* in both mechanisms (Table 2, Figure 4).

The meroditerpene epitaondiol (**2**) also showed virucidal activity (99.27% of inhibition) and it was also capable of inhibiting the penetration of viral particles into cells. However, unlike the other two substances, it had no activity in the post-penetration stages. As observed with other substances, epitaondiol was not able to interact with the cellular receptors.

The methyl ester obtained from atomaric acid showed different properties from those observed for the parent atomaric acid. It showed better extracellular activity (99.9% of inhibition against 61.2% of atomaric acid), which can be explained by the higher lipophilicity of the methyl ester, when compared to the acid (logP 6.32 and 2.64, respectively). It was also capable of inhibiting penetration of viral particles (51.4% of inhibition while no activity was observed for atomaric acid), although it has not shown activity in the post-penetration events and cellular receptors.

For comparison purposes, the anti-HMPV activity of ribavirin (1-beta-D-ribofuranosyl-1*H*-1,2,4-triazole-3-carboxamide) was evaluated. Ribavirin is an antiviral drug approved by the American Food and Drug Administration (FDA) for the treatment of RSV infections. However, its efficacy is limited, and its use remains a controversial issue [33]. The antiviral activity of ribavirin related to inhibition of HMPV replication is equivalent to that observed with RSV [18]. In the present study, ribavirin only showed post-penetration activity, which corroborates its described mechanism of action [34].

In a previous study we have demonstrated the anti-HMPV activity of the marine alga *Ulva fasciata* [35]. In that study we showed the effect of variable environment and chemical procedures used to obtain the extract on its biological properties. In this work, we demonstrated that substances obtained from *S. zonale* also possess anti-HMPV activity and that this activity is closely related to the molecular structures.

## 4. Experimental

### 4.1. General

Analytical thin-layer chromatography (TLC) separations were carried out on Merck silica gel 60 F-254 (0.2 mm) precoated aluminum plates. Once developed, plates were visualized by spraying with 2% ceric sulphate in sulfuric acid, followed by gentle heating. Silica gel 60 (Merck, 70–230 and 230–400 mesh) was used for column chromatography. ^1^H-NMR and ^13^C-NMR, HMQC, HMBC, and COSY spectra were measured employing a Varian 300 instrument operating at 300 MHz for ^1^H-NMR and at 75 MHz for ^13^C-NMR, using TMS as internal standard. EI-MS spectrum was taken on a Sync VG Autospec spectrometer at 70 eV. 

### 4.2. Sample Collection

Specimens of *S. zonale* were collected at Praia do Forno, Município de Búzios, Rio de Janeiro State (22°45′ S, 41°52′ W), Brazil in February 2007. The seaweed was washed with local sea water, separated from sediments, epiphytes and other associated organisms. The algae were identified by Dr. Lísia M. Gestinari. Voucher specimen was deposited at the herbarium of the Universidade Federal do Rio de Janeiro (RFA 3823).

### 4.3. Extraction and Isolation

Air-dried alga (130.0 g dry weight) was extracted with dichloromethane (2 L × 3 times, at room temperature for 3 weeks) and concentrated to give a dark brown oil (11.8 g). A portion of the crude extract (2.5 g) was subjected to a series of column chromatographies on silica gel to give the meroditerpenoids atomaric acid (**1**, 0.006 g) and epitaondiol (**2**, 0.003 g). The chemical structure of these compounds was established by ^1^H-NMR and ^13^C-NMR spectral data analysis and comparison of the spectroscopic data with reported values [24,31]. The methyl ester of atomaric acid was obtained from structural modification. Briefly, a portion of the crude extract (1.0 g) was subjected to a methylation reaction performed by dissolving a portion of the crude extract in CHCl_3-_MeOH (4:1) and adding fresh diazomethane (CH_2_N_2_) in a solution of ethyl ether in excess. The mixture was left overnight under magnetic stirring [35]. Next, it was subjected to successive column chromatographies to yield atomaric acid methyl ester (**3**, 0.136 g).

*Atomaric acid methyl ester* (**3**) was obtained as yellow oil: ^1^H-NMR (CDCl_3_, 300 MHz) δ: 0.93 (s, 3H, H-16), 1.02 (s, 3H, H-17), 1.15 (d, 3H, *J* = 8.0 Hz, H-15), 1.26 (m, 1H, H-4a), 1.38 (m, 1H, H-7), 1.49 (m, 2H, H-5), 1.51 (m, 1H, H-8b), 1.57 (m, 1H, H-12a), 1.66 (s, 3H, H-20), 1.68 (s, 3H, H-19), 1.73 (m, 1H, H-3), 1.74 (m, 1H, H-8a), 1.81 (m, 1H, H-12b), 1.88 (m, 1H, H-4b), 1.96 (m, 1H, H-9a), 2.22 (s, 3H, H-7´), 2.26 (m, 2H, H-13), 2.32 (m, 1H, H-11), 2.39 (m, 1H, H-9b), 2.41 (d, 1H, *J* =14.0, H1a), 2.84 (d, 1H, H-1b), 3.72 (s, 3H, 8’-OCH_3_), 3.65 (s, 3H, -COOCH_3_), 4.27 (sl, -OH), 6.54 (d, 1H, *J* = 3.00 Hz, H-4’), 6.69 (d, 1H, *J* = 3.00 Hz, H-2’). ^13^C-NMR (CDCl_3_) δ: 15.7 (C-15), 16.8 (C-17), 17.9 (C-7´), 20.4 (C-16, 19), 20.7 (C-20), 22.2 (C-8), 23.3 (C-9), 25.0 (C-4, 12), 35.6 (C-1, 3), 33.0 (C-13), 36.6 (C-5), 38.9 (C-6), 40.6 (C-2), 41.9 (C-7), 51.4 (-COOCH_3_), 53.1 (C-11), 55.5 (8´-OCH_3_), 113.2 (C-4´), 114.7 (C-2’), 123.2 (C-10), 124.0 (C-6´), 126.8 (C-3´), 133.0 (C-18), 146.8 (C-1´), 152.6 (C-5´), 174.7 (C-14).

### 4.4. Cell Culture

LLC-MK2 cells (*Macaca mulatta* [monkey, rhesus]) were grown in Dulbecco’s modified Eagle’s medium (DMEM), supplemented with 3 mM L-glutamine, 50 mg/mL garamicin, 2.5 mg/mL fungizon, sodium bicarbonate at 0.25%, and 10% of heat-inactivated fetal bovine serum (FBS) and maintained at 37 °C in an atmosphere of 5% CO_2_. All experiments were carried out using the same amount of cells (5 × 10^5^ cells/mL).

### 4.5. Virus

A sample of HMPV NL/1/00 was kindly provided by ViroNovative BV (Erasmus University, Rotterdam, The Netherlands). Because the viral replication *in vitro* is dependent of trypsin, this enzyme was added to the culture medium for a final concentration of 1 μg/mL in all the antiviral and cytotoxicity experiments.

### 4.6. Determination of Cytotoxicity

The cytotoxicity assay was performed by incubating LLC-MK2 cell monolayers cultivated in 96-well microplates with two-fold serial dilutions of the compounds in triplicate, for seven days at 34 °C. The cellular viability was further evaluated by the neutral red dye-uptake method [37].

### 4.7. Antiviral Assay

LLC-MK2 cell monolayers cultivated in 48 well-microplates were treated with the compounds at the concentration chosen according to the cytotoxicity results. The wells reserved for cell culture and virus control were not treated with the compounds. Afterwards, a HMPV suspension (100 μL) diluted at 10^−1^ corresponding to 1.12 × 10^7^ copies/mL, was added to treated and untreated cell cultures and incubated in a 5% CO_2_ atmosphere at 34 °C for seven days. All experiments were carried out in triplicate. After incubation the supernatant of the cell monolayers was removed and then lysated using guanidine thiocyanate buffer. The viral RNA was extracted and a real time RT-PCR for detections of HMPV genome was performed using primers that amplify a 151 bp fragment of the HMPV N gene, as previously described by Brittain-Long *et al.* [38]. The antiviral activity was evaluated comparing the number of copies of viral genome obtained from the supernatant of the cell cultures in the presence of the substances with that of the virus control that was inoculated in the same plate in an untreated cell culture.

### 4.8. Extraction of Viral RNA

Viral RNA was extracted from the cells supernatant lysate using the commercial kit Totally RNA™ (Applied Biosystems/Ambion, USA), according to the manufactory’s instructions.

### 4.9. Real-Time RT-PCR

Reverse transcription was performed on a reaction mixture (10 μL) containing extracted viral RNA (5 μL) and 360 nM of the antisense primer, using ImProm-II Reverse Transcriptase (Promega, Madison, WI, USA). Real-time PCR assays were performed in a Step-One Real Time PCR System (Applied Biosystems, Carlsbad, CA, USA) and consisted of 5 min activation at 94 °C, followed by 45 cycles of 10s at 94 °C, 10s at 60 °C and 10s at 72 °C. Amplification was carried out on 24 μL reaction volumes, including 5 μL of cDNA, 900 nM of sense primer and 300 nM of antisense primer, and 12 μL of Power SYBR-Green qPCR mix (Ludwig Biotecnologia, Alvorada, RS, Brazil). The quantification of HMPV RNA was performed using a standard curve generated by the Ct (threshold cycle) values obtained from serial 10-fold dilutions of *in vitro* transcripts containing dilutions varying from 10^0^ to 10^7^ of the original stock of virus. Each dilution was quantified using the Quant-IT™ RNA Assay Kit (Invitrogen, Carlsbad, CA, USA), which allows us to correlate the amount of RNA, *i.e.*, the number of genome copies, with the Ct of each dilution. The results were analyzed in triplicate, and the average number of copies of the viral RNA was calculated.

### 4.10. Determination of the Mechanisms of Action

Once the ability of the compounds to inhibit the replication of HMPV was established, several experiments were carried out to elucidate the mechanism involved in its antiviral activity. The experiments were designed in order to demonstrate whether the activity was on the viral particle (virucidal), on the virus-cell interaction (receptors and cell entry) or in a late stage of virus replication (intracellular activity). 

#### 4.10.1. Virucidal Assay

One hundred microliters of a HMPV suspension diluted at 10^−1^ was added to either 900 μL of the compounds or DMEM-Eagle without serum (control), according to Chen *et al.* [39]. All mixtures were incubated at 37 °C for two hours and, immediately after, they were inoculated in LLC-MK2 cell monolayers grown in 48-well plates, which were incubated for seven days at 34 °C in atmosphere of 5% CO_2_. After incubation the supernatant of the cell monolayers was removed, and then lysated using guanidine thiocyanate buffer. The viral RNA was extracted and real time RT-PCR for HMPV was performed as previously described.

#### 4.10.2. Cellular Receptors Assay

In order to evaluate the possible effect of the compounds on cell receptors, they were added to LLC-MK2 cell monolayers, incubated at 4 °C for one hour, washed three times with cold DMEM-Eagle and 100 µL of a HMPV suspension diluted at 10^−1^ was added to treated and untreated cell culture and incubated at 34 °C for seven days. After incubation the supernatant of the cell monolayers was removed and lysated using guanidine thiocyanate buffer. The viral RNA was extracted and Real Time RT-PCR for HMPV was performed as previously described.

#### 4.10.3. Cell Entry Assay

LLC-MK2 cell monolayers were inoculated with 100 µL of a HMPV suspension diluted at 10^−1^ and incubated for one hour at 4 °C. After absorption of the inocula, the monolayers were washed, treated with the compounds, followed by incubation for one hour at 37 °C. Afterwards, the monolayers were washed, DMEM-Eagle was added and the cells were incubated at 34 °C for seven days in an atmosphere of 5% CO_2_. After incubation the supernatant of the cell monolayers was removed and lysated using guanidine thiocyanate buffer. The viral RNA was extracted and Real Time RT-PCR for HMPV was performed as previously described.

#### 4.10.4. Intracellular Assay

LLC-MK2 cell monolayers were inoculated with 100 μL of a HMPV suspension diluted at 10^−1^ and incubated at 37 °C for two hours. After incubation, the cell monolayers were washed and the substances added. Then, the cells were incubated at 37 °C for 10 h. After incubation the supernatant of the cell monolayers was removed and these were washed with culture medium and lysated using guanidine thiocyanate buffer. The viral RNA was extracted and Real Time RT-PCR for HMPV was performed as previously described.

#### 4.10.5. Molecular Modeling

Structures were built and energy-minimized using the software Marvin (http://www.chemaxon.com/ products/marvin/) and MOPAC 2007 [40]. Next, logP and molecular surface area (solvent-accessible) were calculated for neutral and ionized species at pH 7.4, when necessary using the software Marvin.

## 5. Conclusions

In summary, our study has demonstrated that the crude extract and the meroditerpenoids epitaondiol, atomaric acid and its methyl ester derivative isolated from the marine seaweed *S. zonale* possess strong anti-HMPV activity. Unlike atomaric acid and its methyl ester derivative, the meroditerpene epitaondiol (**2**) was capable of inhibiting the penetration stage into cells of the viral replication, even though it has shown higher cytotoxicity, probably due to particular molecular recognition events associated to its biological mechanism of action that may be further explored. Taken together, these studies confirm the enormous potential of Brazilian marine algae as a source for biomolecules and also suggest that bio-guided assays are a valuable tool in order to assist the identification of potent bioactive compounds.

## Figures and Tables

**Table 1:** Correlation between cytotoxicity and antiviral activity.

Compound	Concentration (µg/mL)	CC_50_ (µg/mL)	Inhibition (%)	ED_50_ (µg/mL)	SI
*S. zonale* crude extract	12.5	30.76	97.7	1.48	20.78
Atomaric Acid (**1**)	25	>200	99.4	3.52	>56.81
Epitaondiol (**2**)	12.5	49.76	99.9	1.01	49.26
Atomaric acid methyl ester (**3**)	12.5	34.12	99.9	2.66	12.82
Ribavirin	50	>200	99.9	1.68	>119.04

*CC_50_* 50% cytotoxic concentration, *ED_50_* effective dose to reduce virus titers by 50%, *SI* selectivity index.

**Table 2:** Mechanism of action of the compounds.

Compound/Stages of viral replication	Virucidal *	Receptors *	Penetration *	Post-penetration *
*S. zonale* crude extract	95.7	NA	NA	99.9
Atomaric Acid (**1**)	61.2	NA	NA	76.9
Epitaondiol (**2**)	98.5	NA	99.9	NA
Atomaric acid methyl ester (**3**)	99.9	NA	51.48	NA
Ribavirin	NA	NA	NA	99.9

* Results in percentage of inhibition; NA – No activity.
